# Role of FGF System in Neuroendocrine Neoplasms: Potential Therapeutic Applications

**DOI:** 10.3389/fendo.2021.665631

**Published:** 2021-04-14

**Authors:** Giovanni Vitale, Alessia Cozzolino, Pasqualino Malandrino, Roberto Minotta, Giulia Puliani, Davide Saronni, Antongiulio Faggiano, Annamaria Colao

**Affiliations:** ^1^ Laboratory of Geriatric and Oncologic Neuroendocrinology Research, Istituto Auxologico Italiano, IRCCS, Cusano Milanino, Italy; ^2^ Department of Medical Biotechnology and Translational Medicine, University of Milan, Milan, Italy; ^3^ Department of Experimental Medicine, Sapienza University of Rome, Rome, Italy; ^4^ Endocrinology, Department of Clinical and Experimental Medicine, Garibaldi-Nesima Medical Center, University of Catania, Catania, Italy; ^5^ Department of Clinical Medicine and Surgery, Federico II University of Naples, Naples, Italy; ^6^ Oncological Endocrinology Unit, IRCCS Regina Elena National Cancer Institute, Rome, Italy; ^7^ Department of Clinical and Molecular Medicine, Sapienza University of Rome, Rome, Italy

**Keywords:** neuroendocrine neoplasms, FGFR (fibroblast growth factor receptor), FGF (fibroblast growth factor), VEGF - vascular endothelial growth factor, VEGFR - vascular endothelial growth factor receptor

## Abstract

Neuroendocrine neoplasms (NENs) are a heterogeneous group of tumors originating from neuroendocrine cells dispersed in different organs. Receptor tyrosine kinases are a subclass of tyrosine kinases with a relevant role in several cellular processes including proliferation, differentiation, motility and metabolism. Dysregulation of these receptors is involved in neoplastic development and progression for several tumors, including NENs. In this review, we provide an overview concerning the role of the fibroblast growth factor (FGF)/fibroblast growth factor receptor (FGFR) system in the development and progression of NENs, the occurrence of fibrotic complications and the onset of drug-resistance. Although no specific FGFR kinase inhibitors have been evaluated in NENs, several clinical trials on multitarget tyrosine kinase inhibitors, acting also on FGF system, showed promising anti-tumor activity with an acceptable and manageable safety profile in patients with advanced NENs. Future studies will need to confirm these issues, particularly with the development of new tyrosine kinase inhibitors highly selective for FGFR.

## Introduction

Neuroendocrine neoplasms (NENs) are a heterogeneous group of tumors originating from neuroendocrine cells dispersed in different organs (1–5).

Receptor tyrosine kinases are a subclass of tyrosine kinases with a relevant role in several cellular processes including proliferation, differentiation, motility and metabolism. Dysregulation of these receptors plays a relevant role in neoplastic development and progression for several tumors, including NENs (6, 7).

In this review, we provide an overview concerning the role of the fibroblast growth factor (FGF)/fibroblast growth factor receptor (FGFR) system in NENs.

## FGF System in Health and Cancer

FGFs and related receptors are members of a large family with a wide range of effects. This system is involved in organogenesis (during development), homeostasis and repair of adult tissues. Moreover, FGF family promotes angiogenesis, growth, differentiation and migration of cells mainly through the activation of RAS-MAPK, PI3K-AKT and PLCγ pathways, with a relevant role in the development and progression of several tumors (8). These effects are mediated by the interaction of FGFs with four tyrosine kinase receptors: FGFR1, FGFR2, FGFR3 and FGFR4, which are composed by an extracellular domain, a transmembrane domain and an intracellular domain. The binding of ligands induces conformational changes that lead to a dimerization of these receptors. This event activates the intracellular tyrosine kinase domain, which in turn triggers the signalling cascade (8). FGFs, based on their biochemical functions, sequence similarity and evolutionary relationships, are classified into different subfamilies: FGF1, FGF4, FGF7, FGF8, FGF9, FGF15/19 and FGF11.

FGF1 and FGF2 are members of the FGF1 subfamily. FGF1 is the only FGF that can activate all FGFRs splice variants. It is involved in cell cycle regulation, cell differentiation, survival and apoptosis. FGF1 plays a central role in neuroprotection and axon regeneration and appears to improve functional recovery after spinal cord injury (9). FGF2 has known angiogenic properties (10, 11). The FGF4 subfamily (FGF4, 5,6) can activate FGFR1-3(IIIc) and FGFR4. These molecules are fundamental in embryonic development and muscle regeneration (8, 9). FGF7 subfamily (FGF3, 7, 10, 22) preferentially activates FGFR2(IIIb), although FGF3 and FGF10 can also interact with FGFR1(IIIb). FGF3 is involved in the neural development, while FGF7 is required for lung, kidney and neuronal synapses development. The development of epithelial components, such as limb and lungs, and mammary gland requires epithelial-mesenchymal interactions granted by FGF10. Finally, FGF22 regulates the circuit remodeling in the injured spinal cord (12–15). FGF8 subfamily members (FGF8, 17 and 18) activate FGFR4 and FGFR1-3(IIIc). They are involved in the skeletal and brain development and in odontogenesis (8, 14, 16–18). The FGF9 subfamily (FGF9, 16, 20) interacts with FGFR1-3(IIIc), FGFR3(IIIb) and FGFR4. These proteins are involved in a proper heart, kidney and skeletal development (8, 14, 19, 20). The FGF15/19 subfamily comprises FGF15/19, 21 and 23. FGF15/19 bind FGFR1-3(IIIc) and FGFR4. FGF21 can activate FGFR1(IIIc) and 3(IIIc), as well as FGF23, which can also interact with FGFR4. This subfamily acts as hormones and regulates hepatocyte and adipocyte metabolism (8, 14). FGF11 subfamily members (FGF11, 12, 13, 14) are known as intracellular FGFs. These peptides are not secreted and interact with the cytosolic carboxy terminal tail of voltage gated sodium channels. They cover an important role in the development of the nervous system (8, 21).

A deregulation of the FGF/FGFR system can be involved in cancer development and progression through modulation of cell proliferation, migration and angiogenesis (22).

Besides its role in physiological angiogenesis, FGF2 is implied in tumor-induced angiogenesis and metastatic process and appears to direct tumor-associated macrophages toward a pro-tumorigenic state (23–25). FGF4 promotes cancer cell proliferation, invasion and migration by causing a switch of the receptor FGFR2-IIIb, a splice variant expressed in epithelial cells, into FGFR2-IIIc, expressed in mesenchymal cells and able to induce epithelial-mesenchymal transition (26). FGF5 can promote osteosarcoma proliferation by activating the MAPK signaling pathway (27) and the FGF5/FGFR1 axis contributes to melanoma progression (28). FGF6 can stimulate proliferation of prostate cancer cells through the activation of FGFR4 (29). Among the FGF7 subfamily, FGF3 and FGF7 have been reported to be highly expressed in breast cancer (30, 31) and gastric adenocarcinoma (32), respectively. In addition, the FGF10/FGFR-IIb signaling appears to have a role in breast and pancreatic tumors (15, 33). Although the mechanism is unclear, Jarosz et al. observed a potential role of FGF22 in skin tumorigenesis (34). In a recent study, FGF22 and its receptor FGFR-IIb appear to be associated with the development of lung adenocarcinoma through the MAPK and Rap I signaling pathways (35). A deregulation of FGF18, caused by an altered expression of its negative regulator miR-590-5p, is able to stimulate proliferation and epithelial-mesenchymal transition, with enhanced invasion abilities, in gastric cancer cells (36). In HER^+^ breast cancer cell lines, overexpression of FGF18 stimulates the expression of genes involved in migration and cancer metastasis through Akt/GSK3β pathway (37). By the interaction with FGFR2 and FGFR3 and the activation of the ERK/Akt pathway, FGF18 is able to induce proliferation and invasion in endometrial carcinoma (38).

The FGF/FGFR pathway has also a key role in the onset of drug-resistance (39). FGF/FGFR pathway is the first compensatory mechanism in tumors resistant to drugs targeting the vascular endothelial growth factor (VEGF) system (40–42). Indeed, VEGF-dependent vessels are suppressed during prolonged anti-VEGF therapy, while the expression of FGF2 is increased, leading to a novel angiogenesis dependent on FGF2 signaling pathway. This condition drives the tumor toward drug-resistance (42). Boichuk et al. (43) showed that FGF signaling is activated in gastrointestinal stromal tumors after the acquisition of imatinib resistance. Interestingly, the use of a potent FGF inhibitor markedly reduced cell growth in resistant cells compared to imatinib-sensitive cells. This effect increased when the two molecules were combined in resistant cells, showing also that the FGF-inhibitor can restore sensitivity to imatinib.

## FGF System in Neuroendocrine Neoplasms

The role of the FGF/FGFR system has been analyzed also in NENs and several lines of evidence support its function in the modulation of tumor fibrosis, proliferation, angiogenesis and drug resistance, through a dynamic cross talk between NEN cells, fibroblasts, endothelial cells and inflammatory cells (44).

Bordi et al. identified FGF2 by immunohistochemistry in endocrine cells of the gastric oxyntic mucosa and mRNA of FGF2 in enterochromaffin-like carcinoid tumors (45). Immunohistochemical studies demonstrated the staining for FGF-2 in 100% of NEN cells from the midgut and the pancreas, while FGF2 receptors were observed only in the stromal component (46). La Rosa et al. found cytoplasmic immunoreactivity for FGF1 in 26 (43%) out of 60 GEP-NENs and FGFR1-4 were found in 68-88% of tumors with tumor microenvironment components also expressing FGFRs (47). The authors observed also that normal endocrine cells of the gut rarely expressed FGFRs thus hypothesizing that in normal mucosa the FGF/FGFR system has not an autocrine role on modulating endocrine cells functions. Therefore, *de novo* expression of FGFRs by NEN cells may play a role in the autocrine/paracrine signaling responsible of tumorigenesis, stromal fibrosis and tumor-induced angiogenesis.

NEN are often characterized by the development of fibrosis, local or distant. The best-known fibrotic complications are carcinoid heart disease, which develops in about 20% of patients with carcinoid syndrome (48), and mesenteric fibrosis, which affects up to 40-50% of small bowel NENs (49, 50). Less known complications are represented by retroperitoneal fibrosis (50), scleroderma (51), infiltration of the pleura (52) or alveoli (53) and fibrosis of the bladder (54). Although the pathogenesis of fibrotic complications is unclear, serotonin, with a relevant mitogenic power on fibroblasts, mesangial cells, smooth muscle cells, endothelial cells and NEN cells, may have a role in these events (55). The FGF system appears to be also implicated in the mechanism of gastrointestinal NEN fibrosis (56). In fact, Bordi (45) showed that among the 10 patients suffering from type 3 gastric NEN and with positive immunohistochemistry for FGF2, some had diffuse stromal fibrosis. Another study (57), which analyzed a pool of 41 gastrointestinal NENs, showed a positive correlation between FGF1 and the amount of fibrous stroma in tumors. The FGF is responsible of cell proliferation and stroma formation and its action is potentiated by serotonin (58). Moreover, FGF may activate also the expression of the connective tissue growth factor genes that regulate myofibroblast differentiation, collagen synthesis and fibroblast proliferation (59).

The mRNA expression of FGF receptor was found more frequently in functioning NENs (including gastrinomas and insulinomas) than in functionally inactive NENs (53.6% vs. 22.2%) (60). Although this difference was not statistically significant (p=0.10), speculating on the association between FGFR expression and hormone production may be not totally irrational, but further evidence is required to corroborate these findings.

The FGFR4-G388R single-nucleotide polymorphism was investigated in 71 patients with pancreatic NEN (61). The authors observed that FGFR4-R388 allele was independently associated with liver metastases. To further analyze the impact of the FGFR4 SNP, the same authors transfected BON1 cells with either FGFR4-G388 or FGFR4-R388 and injected them in SCID mice. They found that xenografts expressing FGFR4-R388 displayed a more aggressive biological behavior and were resistant to everolimus treatment. This latter aspect was investigated also among 17 patients previously treated with everolimus in a clinical trial. Patients harboring FGFR4-R388 allele achieved a worse tumor response (9% vs. 25%) and a reduced median PFS (4.8 vs. 16.6 months) and OS (9.3vs 40 months) compared to patients homozygous for FGFR4-G388. Although decreased drug response was related to persistently high mTOR and STAT3 phosphorylation despite of everolimus treatment, these data were not confirmed by Cros et al., who reported no modification of the mTOR pathway in patients with pancreatic or ileal NENs harboring FGFR4-R388 allele (62). This apparent inconsistency corroborates the need for further studies validating the identification of molecular parameters useful to predict drug efficacy and resistance (63).

The FGF/FGFR system collaborates with the VEGF signaling pathway in the initiation and maintenance of tumor angiogenesis. These mechanisms have been demonstrated in allograft transplantation experiments and in mouse model of pancreatic NEN (the Rip1Tag2 transgenic mice), where interfering with the FGF function by a soluble form of the FGFR2 IIIb significantly inhibited tumor-induced angiogenesis and tumor growth (64). The FGF system acts as a second proangiogenic circuit, indeed VEGF is the main regulator of angiogenesis but, as reported by Casanovas et al., experiments in the Rip1Tag2 model of pancreatic islet carcinoma documented that initial inhibition of the angiogenesis achieved by VEGF signaling blockade was restored by the upregulation of the FGF system (65). Therefore, blocking both VEGF and FGF signaling pathways may reveal synergic antiangiogenic effects and inhibit tumor progression secondary to compensatory feedback loops driving tumor revascularization. For instance, Allen et al. investigated the effect of brivanib, a selective inhibitor targeting both VEGF and FGF receptors, in a mouse model of pancreatic NEN. Brivanib was effective not only as first-line therapy, but also as second-line treatment after failure of two agents inhibiting VEGF receptors (DC101 and sorafenib) (66).

## FGFRs as Therapeutic Target in NENs

In the last few years, the therapeutic approach for NENs has changed following the approval of several innovative targeted treatments such as tyrosine kinase inhibitors (TKIs). Although no specific FGFR kinase inhibitors have been evaluated in NENs, several clinical trials on multitarget TKIs, acting also on FGF, are ongoing and few published studies have demonstrated their efficacy in NENs (44). The interest in FGF pathway inhibitors relies also in the possibility to overcome resistance to VEGF inhibition that may arise after long term use of these drugs or could be intrinsic in tumor expressing FGF2 (67–69). The results of clinical trials in NENs evaluating multitarget TKI, acting also on FGF, are described below (**Table 1**).

**Table 1:** Clinical trials evaluating the effects of multitarget tyrosine kinase inhibitors, acting also on FGFR, in patients with NENs.

Ref	Therapy and dose	Molecular target	Study design (Trial name)	Tumors	Number of patients(placebo)	Median follow- up(placebo)	Primary outcome	Results	Main AE (%)
(70)	Surufatinib 300 mg/day	VEGFR 1,2,3 FGFR1 CSF-1R	Randomised, double-blind, placebo-controlled, phase 3 (SANET-EP)	Advanced extrapancreatic NETs (G1-G2)	129 (69)	13.8 months (16.6 months)	PFS	Median PFS: 9.2 months (surufatinib) vs. 3.8 months (placebo)	Hypertension (36%); proteinuria (19%)
(71)	Surufatinib 300 mg/day	VEGFR 1,2,3 FGFR1 CSF-1R	Randomised, double-blind, placebo-controlled, phase 3 (SANET-P)	Advanced pancreatic NETs (G1-G2)	113 (59)	19.3 months (11.1 months)	PFS	Median PFS: 10.9 months (surufatinib) vs. 3.7 months (placebo)	Hypertension (38%); proteinuria (10%); hypertriglyceridemia (7%)
(72)	Surufatinib 300 mg/day	VEGFR 1,2,3 FGFR1 CSF-1R	Dose escalation/expansion study	Heavily pre-treated progressive NETs	32	19 weeks	ORR	9.4%	Hypertension, fatigue, diarrhea
(73)	Surufatinib 300 mg/day	VEGFR 1,2,3 FGFR1 CSF-1R	Phase 2, open label, two stage design study	Advanced MTC	27	–	ORR	22.2%	hypertension (20.3%), proteinuria (11.9%), hypertriglyceridemia (5.1%)*
(74)	Lenvatinib 24 mg/day	VEGFR 1-3 FGFR1-4	Prospective multicohort phase 2 (TALENT)	Advanced pancreatic and gastrointestinal NETs (G1-G2)	111	19 months	ORR	42.3% pancreatic 16.3% gastrointestinal	Hypertension (22%); fatigue (11%); diarrhea (11%)
(75)	Lenvatinib 24 mg/day	VEGFR 1-3 FGFR1-4	Phase 2, multicenter, open-label, single-arm clinical trial	Unresectable or metastatic progressive MTC	59	–	ORR	36% (all PR)	Diarrhea (14%); hypertension (7%); decreased appetite (7%)
(76)	Lenvatinib 24 mg/day	VEGFR 1-3 FGFR1-4	Nonrandomized, open-label, multicenter, phase 2 study	Progressive MTC	9	9.6 months	Safety	100% of patients ≥1 AE; 1.7% of patients AE leading to discontinuation	Decreased appetite (100%); hypertension (89%); palmar-plantar erythrodysesthesia (89%)
(77)	Lenvatinib 24 mg/day	VEGFR 1-3 FGFR1-4	Prospective, post-marketing observational study	UnresectableMTC	28	12 months	Safety	100% pts ≥1 AE	Hypertension; proteinuria; palmar-plantar erythrodysesthesia
(78)	Nintedanib	VEGFR 1,2,3 FGFR2	Multicenter phase 2 study	Advanced progressing carcinoid on stable dose SSA for ≥3 months	30	16 weeks	PFS	PFS at 16 weeks 86.7% in 26 pts	Diarrhea (18%); increase in GGT (18%); lymphopenia (18%)
(79)	Anlotinib 12 mg/day	VEGFR 2-3 FGFR1-4	Single-arm phase 2 study	Advanced or metastatic MTC	58	9.8 months	PFS	PFS at 48 weeks 84.5%	Hand-foot syndrome (79.3%); hypertriglyceridemia (46.5%); elevated cholesterol levels (43.1%)
(80)	Anlotinib 12 mg/day	VEGFR 2-3 FGFR1-4	Multicenter, randomized, double-blind, placebo-controlled phase IIB trial (ALTER01031)	Advanced or metastatic MTC	62 (29)	–	PFS	Median PFS: 20.67 months (anlotinib) vs 11.07 months (placebo)	Hand-foot syndrome; hypertension; hypertriglyceridemia

AE, adverse events; FGFR, fibroblast growth factor receptor; MTC, medullary thyroid carcinoma; NET, neuroendocrine tumor; ORR, overall response rate; PFS, progression free survival; pts, patients; SSA, somatostatin analogs; VEGFR, Vascular Endothelial Growth Factor Receptor.

*data reported for the overall population (differentiated thyroid cancer and MTC).

*Surufatinib* is a potent TKI targeting VEGF receptors (VEGFR) 1, 2, and 3, FGFR1, and CSF-1R. In preliminary phase I and Ib/II studies surufatinib showed encouraging anti-tumor activity in advanced NENs (81, 82).

Two randomized phase III placebo controlled trials evaluated safety and efficacy of surufatinib in patients with well differentiated NENs of extra-pancreatic (SANET-ep) and pancreatic (SANET-p) origin (70, 71).

In SANET-ep study (70) 198 patients were randomly assigned to surufatinib 300 mg/day (n=129) or placebo (n=69). Median progression-free survival (PFS) was 9.2 months in the surufatinib group versus 3.8 months in the placebo group. The overall response rate (ORR) was 10% in the surufatinib group versus zero in the placebo group. The most common treatment-related adverse events (AE) of grade ≥ 3 were hypertension (36% surufatinib vs 13% placebo) and proteinuria (19% vs. 0%). In SANET-p study (71) 113 patients were randomly assigned to surufatinib (300 mg/day) and 59 to placebo. The median PFS was 10.9 months for surufatinib versus 3.7 months for placebo; ORR was 19% in the surufatinib group and 2% in the placebo group. The most common AE of grade ≥ 3 were hypertension (38% surufatinib *vs.* 7% placebo), proteinuria (10% *vs.* 2%) and hypertriglyceridemia (7% *vs.* none).

Another study evaluated the effect of surufatinib dose escalation/expansion in 32 patients with heavily pre-treated progressive NENs, 16 patients with pancreatic NENs and 16 with extra-pancreatic NENs. Nineteen patients remained on active treatment (13 extra-pancreatic and 6 pancreatic), 9 patients discontinued due to disease progression, 2 withdrew consent and 2 discontinued due to AE. An ORR of 9.4% was observed (72).

An open label phase II study evaluated efficacy and tolerability of surufatinib (300 mg/day) in 27 patients with progressive medullary thyroid cancer (MTC). Objective response was observed in 22.2% of patients with MTC, and the majority (88.9%) achieved disease control. The therapy was well tolerated (73).

Therefore, surufatinib demonstrated promising anti-tumor activity with an acceptable and manageable safety profile in advanced NENs.

*Lenvatinib* is a potent VEGFR1-3 and FGFR1-4 inhibitor. The TALENT trial, a prospective phase II study, evaluated efficacy, safety and tolerability of lenvatinib (24 mg once daily) in G1/G2 advanced pancreatic (n=55) and gastrointestinal (n=56) NENs resistant to previous targeted agents. The ORR was 29% (42.3% for pancreatic NENs and 16.3% for gastrointestinal NENs). PFS and overall survival (OS) for pancreatic NENs were 15.5 months and 29.2 months, while for gastrointestinal NENs were 15.4 months and not reached, respectively. The most frequent grade 3/4 AE were hypertension (22%), fatigue (11%) and diarrhea (11%) (74). Thus, lenvatinib showed a promising PFS and OS in a pretreated population.

A phase II, multicenter, open-label, single-arm clinical trial evaluated efficacy and tolerability of lenvatinib (24-mg daily, 28-day cycles) in 59 patients with MTC. ORR was 36%, all PR. Disease control rate (DCR) was 80%, 44% had SD. Median time to response was 3.5 months. Median PFS was 9.0 months. Grade 3/4 AE included diarrhea (14%), hypertension (7%), decreased appetite (7%), fatigue, dysphagia and increased alanine aminotransferase levels (5% each) (75).

Another phase II study evaluated lenvatinib treatment in 9 patients with MTC. The most frequently reported AE were decreased appetite (100%), hypertension (89%), palmar-plantar erythrodysesthesia (89%), diarrhea (89%), fatigue (78%) and proteinuria (67%). Median PFS was 9.2 months. Median OS was 12.1 months. ORR was 22% and DCR was 100% (76).

Recently, a prospective, post-marketing observational study evaluated, in daily clinical practice, the safety and effectiveness of lenvatinib in 28 patients with MTC. Hypertension, proteinuria and palmar-plantar erythrodysesthesia syndrome were the most frequently reported AE. The 12-months OS rate was 83%. ORR was 45% (77).

*Nintedanib* is a dual inhibitor of VEGFR1, -2, and -3 as well as FGFR2 and showed both antiangiogenic and antitumor activity in the RIP1-Tag2 transgenic mouse model of tumorigenesis for pancreatic NEN (44). A multicenter phase II study evaluated efficacy, safety and tolerability of nintedanib in 30 patients with unresectable/metastatic carcinoids on stable dose of SSA for ≥3 months. PFS at 16 weeks was 86.7% in 26 patients. PR was observed in 4%, SD in 83%, disease progression in 8% of patients. Quality of life was maintained or improved in at least 50% of subjects. The most common grade 3 AE were hypertension and decreased appetite (78).

A prospective randomized double-blind phase II study evaluated the efficacy and tolerability of nintedanib in progressing MTC after prior TKI treatment. The study was stopped due to slow accrual with 32/67 patients enrolled, without reaching the targeted statistical power. The most common AE were diarrhea (18%), nausea (9%), GGT increase (18%) and lymphopenia (18%) (83).

*Anlotinib* is a novel TKI targeting VEGFR2-3 and FGFR1-4 with high affinity. Anlotinib has previously shown promising antitumor activity on MTC in preclinical models and a phase I study (84). A phase II clinical trial showed a relevant antitumor activity of anlotinib (12 mg once daily, two weeks on/one week off) in 58 patients with advanced MTC. PFS rates at 24, 36, and 48 weeks were 92.2%, 87.8% and 84.5%, respectively. Significant decreases in serum calcitonin (≥50%) occurred in 57.5% of patients. The most common AE included hand-foot syndrome (79.3%), hypertriglyceridemia (46.5%), hypercholesterolemia (43.1%), fatigue (41.4%), proteinuria (39.7%), hypertension (39.7%), sore throat (37.9%), diarrhea (34.5%) and anorexia (34.5%) (79).

These data have been confirmed in a phase IIb study (ALTER01031), enrolling a larger cohort of patients (80). Ninety-one patients with advanced MTC were randomized: 62 to anlotinib arm and 29 to placebo arm (12 mg/die from day 1 to 14 of a 21-day cycle). Median PFS was 20.7 months in anlotinib arm vs. 11.1 months in placebo arm. The most common AE after anlotinib arm were hand-foot syndrome, hypertension, hypertriglyceridemia and diarrhea (80).

Several clinical trials on the use of multi target TKI, with an action also on FGFR, in patients with NENs are currently ongoing. **Table 2** reports the main characteristics of trials registered on clinicaltrials.gov.

**Table 2:** Ongoing clinical trials evaluating the effects of multitarget tyrosine kinase inhibitors, acting also on FGFR, in patients with NENs.

Identifier	Therapy	Molecular target	Study design	Tumors	Estimated sample size	Primary outcome	Start date	Estimated Completion Date
*NCT02399215*	Nindetanib	FGFR VEGFR PDGFR	Multicenter open label phase II study	Well or moderately differentiated (G1, G2) NEN not pancreatic	30	PFS	May 2015	October 2020
*NCT04207463*	Anlotinib + AK105 *(anti PD1)*	FGFR VEGFR PDGFR c-kit	Multicenter multi-cohort open label phase II study	G1 or G2 GEP NET (cohort 5)	150 (all cohorts)	ORR	June 2020	December 2020
*NCT02259725*	Regorafenib	FGFR VEGFR1-3 TIE2 KIT RET RAF-1 BRAF BRAFV600E PDGFR	Multicenter multi-cohort open-label phase II study	Carcinoid (cohort A) or pancreatic islet cell tumors (cohort B)	48	PFS	August 2016	August 2021
*NCT03950609*	Lenvatinib + Everolimus *(mTOR inhibitor)*	FGFR1-4 VEGFR1-3	Single center open-label phase II study	Unresectable well differentiated carcinoid tumors	32	ORR	July 2019	May 2021
*NCT03475953*	Regorafenib + Avelumab *(anti PD-L1)*	FGFR VEGFR1-3 TIE2 KIT RET RAF-1 BRAF BRAFV600E PDGFR	Multicenter, open label phase I/II study	G2 or G3 GEP NEN (cohort G)	362	ORR (Phase 2)	May 2018	November 2020
*NCT02657551*	Regorafenib	FGFR VEGFR1-3 TIE2 KIT RET RAF-1 BRAF BRAFV600E PDGFR	Open-label phase II study	Metastatic medullary thyroid cancer	33	PFS	January 2016	October 2022
NCT03008369	Lenvatinib	FGFR1-4VEGFR1-3	Open-label phase II study	Metastatic PPGLs	25	TRR	May 2017	December 2020

FGFR, fibroblast growth factor receptor; GEP, gastro-entero-pancreatic; NA, not available; NEN, neuroendocrine neoplasm; NET, neuroendocrine tumor; ORR, objective response rate; PD1, Programmed cell death protein 1; PDGFR, Platelet-derived growth factor receptor; PD-L1, Programmed cell death ligand 1; PFS, progression free survival; PPGL, Pheochromocytoma and Paraganglioma; pts, patients; TRR, tumor response rate (complete response and partial response); VEGFR, Vascular Endothelial Growth Factor Receptor.

## Conclusions and Future Perspectives

In the last years there is mounting evidence supporting the role of FGF/FGFR system in the development and progression of NENs and probably in the occurrence of fibrotic complications (mesenteric and/or retroperitoneal fibrosis). In addition, the FGF/FGFR pathway could also have a key role in the onset of drug-resistance. Indeed, FGF/FGFR pathway is a main compensatory mechanism in anti-VEGF-therapy-resistant tumors.

Currently no specific FGFR kinase inhibitors have been evaluated in patients affected by advanced NENs. Although recent clinical trials have reported a significant antitumor activity and manageable safety profile of several multitarget TKIs, which are able to block many molecular pathways including FGFR, it is not possible to isolate the efficacy of FGFR inhibition alone. Future studies should better confirm these issues and clarify the role of FGF/FGFR pathway in promoting drug-resistance in NENs. The development of new TKIs, highly selective for FGFR and with less toxicity, may open an innovative therapeutic strategy to be integrated into a personalized approach for this heterogeneous class of tumors. In addition, recent preclinical studies showed a potent inhibition in tumor growth both in hepatocellular carcinoma (85) and in ovarian cancer (86), through the simultaneous blockade of mTOR and FGFR pathways. Considering the pivotal role of deregulated mTOR signaling activation in the proliferation of NENs, particularly in pancreatic tumors, combining mTOR inhibitors and TKIs targeting FGFRs could represent a future therapeutic approach in NENs.

## Author Contributions

GV, AlC, PM, RM, GP, and DS conceptualized and wrote the manuscript. AF and AnC contributed to draft the manuscript. All authors contributed to the article and approved the submitted version.

## Funding

This work was supported by the Italian Ministry of Education, University and Research (MIUR): PRIN 2017Z3N3YC.

## Conflict of Interest

The authors declare that the research was conducted in the absence of any commercial or financial relationships that could be construed as a potential conflict of interest.
